# Oleic Acid and Transferrin Synergistically Induce Serum-Free Adipogenic Differentiation of Porcine Preadipocytes via the SEPTIN4/PPARγ Axis

**DOI:** 10.3390/cells15080684

**Published:** 2026-04-13

**Authors:** Zhou Fu, Yingying Li, Shouwei Wang, Shilei Li, Duo Tang, Xiang Guo, Yu Qi, Pengfei Zhao, Wenting Liu, Chen Guo, Yeting Shen, Feng Yang

**Affiliations:** 1 China Meat Research Center, Beijing Academy of Food Sciences, Beijing 100068, China; fuzhou_tianjin@163.com (Z.F.);; 2 Technology Innovation Center of Animal-Derived Protein Alternatives, State Administration for Market Regulation, Beijing 100076, China; 3 Animal Genetics, Breeding and Reproduction Science, College of Animal Science, Inner Mongolia Agricultural University, Hohhot 010018, China

**Keywords:** cultured meat, serum-free induction, lipid synthesis, oleic acid, transferrin, SEPTIN4, adipogenic differentiation

## Abstract

**Highlights:**

**What are the main findings?**

- A novel serum-free medium (SFM-3) markedly enhances porcine preadipocyte differentiation and lipid accumulation through the synergistic action of oleic acid and transferrin.
- SEPTIN4 promotes porcine adipocyte differentiation via a previously unrecognized activation of the PPARγ/CEBPα axis.

**What are the implications of the main findings?**

- SFM-3 provides a defined strategy to enhance the sensory quality of cultured meat and overcome serum dependency; notably, it elucidates the novel SEPTIN4-PPARγ axis, paving the way for industrialization.

**Abstract:**

Cultured meat represents an emerging frontier in cellular agriculture, garnering increasing interest due to its potential benefits regarding sustainability, animal welfare, and food safety. However, its development is hampered by challenges in flavor modulation and sensory quality, primarily due to the limited biosynthesis of fat-derived flavor compounds. Although adipose tissue engineering has been extensively studied, its industrial-scale production is hampered by serum dependency and low differentiation efficiency. Therefore, the establishment of serum-free, efficient strategies for regulating lipid synthesis is urgently needed. In this study, we developed a serum-free adipogenic induction system and investigated its underlying regulatory mechanisms. We demonstrated that Serum-Free Differentiation Medium 1 (SFM-1) initiated the differentiation program and induced intracellular lipid deposition in preadipocytes (~10% by Day 8). Serum-free differentiation medium 2 (SFM-2), which supplied oleic acid (OA) as a lipid substrate and signaling activator, markedly enhanced lipid droplet accumulation and differentiation efficiency. Ultimately, serum-free differentiation medium 3 (SFM-3), leveraging the synergistic action of oleic acid (OA) and transferrin (TRF), successfully activates the expression of SEPTIN4, which in turn regulates a core adipogenic network—including the master transcription factors PPARγ and CEBPα, as well as downstream functional genes. Mechanistically, the OA/TRF combination in SFM-3 upregulates SEPTIN4, unveiling a previously unrecognized regulatory axis that activates the PPARγ signaling pathway, thereby synchronizing the proliferation and differentiation of precursors and guiding them from initiation to functional maturity. Our study presents a chemically defined, scalable platform for the serum-free adipogenic differentiation of porcine adipocytes, offering a promising strategy for the controllable production of fat components in cultured meat and supporting its industrialization.

## 1. Introduction

Cultured meat has emerged as a promising sustainable protein source [1,2,3,4,5,6]. Significant progress has been made in key areas such as lineage-specific myogenic differentiation, the development of edible scaffolds, and three-dimensional tissue construction, advancing the scalable biomanufacturing of cultured meat [7,8]. However, cultured meat still faces critical challenges in flavor, texture, and nutritional composition [9]. In particular, the precise regulation of adipose tissue and its industrial-scale synthesis remain key bottlenecks in realistic product development [10,11,12]. Intramuscular fat is essential for generating meat flavor precursors (such as aldehydes and ketones). Its content and composition directly determine the juiciness, tenderness, and overall taste richness of meat products [13,14,15,16]. Therefore, achieving the biosynthesis and functional maturation of adipocytes has become a central challenge in enhancing the sensory quality of cultured meat. In recent years, researchers have intensively investigated the in vitro construction of adipose tissue [17]. Efforts include developing animal-component-free or food-grade induction strategies, utilizing edible microcarriers for the large-scale expansion of adipocytes [18], and achieving coordinated structuring of muscle and fat through 3D printing technology [19]. These studies highlight the critical role of fat in the overall sensory properties of cultured meat. However, as key contributors to lipid formation, adipocytes remain challenging to efficiently differentiate in vitro, which limits both the safety and scalability of adipose tissue production for cultured meat.

SEPTIN4, a member of the SEPTIN family, is involved in the construction and maintenance of the cytoarchitecture. Possessing GTPase activity, SEPTIN4 binds and hydrolyzes GTP, playing a key role in intracellular signaling and functional regulation. A study in 2014 found that during the differentiation of adipocyte precursors, the SEPTIN4_I1 isoform decreased while the SEPTIN4_I2 isoform increased [20], suggesting the involvement of SEPTIN4 in the “adipogenic conversion” of cells. However, its specific role in adipogenesis remains unclear.

Research on the in vitro construction of adipose tissue has led to a relatively systematic understanding of the molecular regulatory mechanisms underlying adipogenesis. Existing studies have not only established the central roles of PPARγ and CEBPα in the adipogenic transcriptional cascade [21,22,23,24] but have also elucidated their interconnected regulatory networks and temporal expression patterns. PPARγ is regarded as the “master switch” of adipogenesis [25], and CEBPα forms a positive feedback loop with it, jointly activating downstream genes involved in lipid synthesis and metabolism [26]. In recent years, the application of technologies such as single-cell sequencing and chromatin accessibility analysis has further elucidated the hierarchical relationships within this regulatory network and its underlying epigenetic mechanisms [27,28,29]. However, key bottlenecks remain in applying and adapting tendogenous adipogenic mechanisms to the in vitro adipogenic process [27]. Furthermore, although numerous reports exist on the in vitro synthesis of fat components, the overall synthesis efficiency and stability require further validation. More importantly, this field has long been constrained by its dependence on serum as a key induction factor [30,31]. Current induction systems commonly use animal serum, which has a complex, undefined composition and significant batch-to-batch variation. This leads to unstable differentiation efficiency and carries the risk of exogenous factor contamination [32,33], limiting standardization and industrialization of cultured fat production [34].

Recent studies have indicated that specific metabolic microenvironment components can exert synergistic effects on cell fate regulation that extend beyond their traditional physiological functions. For instance, monounsaturated fatty acids have been confirmed in metabolic studies not only to serve as important precursors for lipid synthesis [35,36] but also to act as endogenous ligands for nuclear receptors like PPARγ, directly participating in transcriptional regulation [37,38,39]. Concurrently, the key iron homeostasis protein transferrin, besides maintaining basic iron metabolism, plays a central role in cell cycle progression and mitochondrial biogenesis [40,41]. Although these functions have been sufficiently elucidated within their respective fields, the roles of different inducing factors in the lipid synthesis process have not been fully elucidated. More importantly, a comprehensive technical system capable of achieving highly efficient in vitro fat biosynthesis has not yet been adequately reported. This makes understanding the key regulatory mechanisms by which crucial lipid synthesis components synergistically regulate transcriptional activation, energy metabolism, and adipocyte development, as well as achieving high-efficiency synthesis, a critical unmet need in fat biosynthesis.

This study aimed to establish a serum-free isolation and adipogenic differentiation system for porcine adipocyte precursors. Through multi-dimensional analyses encompassing cytomorphology, molecular markers, and metabolic activity, we elucidated a mechanism whereby key culture components—oleic acid and transferrin—activate the SEPTIN4/PPARγ/CEBPα axis to drive adipogenic differentiation. This system overcomes the serum dependency bottleneck of existing in vitro adipogenic technologies, establishing an efficient and stable platform for adipogenesis. Our findings provide a theoretical foundation for the controllable construction of adipose tissue in cultured meat and offer critical technical support for enhancing the overall quality and advancing standardized production practices of cultured meat.

## 2. Materials and Methods

### 2.1. Animals and Ethics

One healthy 8-day-old male Beijing Black pig was used for tissue collection. All procedures complied with institutional animal care guidelines. Subcutaneous adipose tissue from the neck region was excised immediately after euthanasia for preadipocyte isolation.

### 2.2. Major Instruments and Equipment

Fluorescence inverted microscope, high-content cell imaging analysis system, Roche LightCycler 480 II (Roche Diagnostics GmbH, Mannheim, Germany) real-time quantitative PCR instrument, chemiluminescence instrument, etc.

### 2.3. Major Chemical Reagents

DMEM medium (Gibco, Grand Island, NY, USA, 6126058), Trypsin (Solarbio, Beijing, China, T1322), Fetal Bovine Serum (FBS) (Gibco, Grand Island, NY, USA, B210865RP), Insulin (INS) (Macgene, Beijing, China, CC101), Dexamethasone (DEX) (MCE, Monmouth Junction, NJ, USA, HY-147220), 3-Isobutyl-1-methylxanthine (IBMX) (MCE, Monmouth Junction, NJ, USA, HY-12318), Rosiglitazone (RSG) (MCE, Monmouth Junction, NJ, USA, HY-17386), Sodium Oleate (NaOL) (Sigma, St. Louis, MO, USA, 143-19-1), 10% PAGE Gel Preparation Kit (Epizyme, Shanghai, China), Protease inhibitor (Epizyme, Shanghai, China, GRF101), RIPA Lysis Buffer (Beyotime, Shanghai, China, P0013B), Oil Red O Staining Kit (Beyotime, Songjiang, Shanghai, China, C0157S), BODIPY 493/503 (Beyotime, Songjiang, Shanghai, China, C2053M), ECL Chemiluminescence Substrate (Meilunbio, Dalian, China, SQ201).

Antibodies: PREF1 (Proteintech, Wuhan, China, 12875-1-AP), APN (Proteintech, China, 66239-1-Ig), FABP4 (Proteintech, Wuhan, China, 67167-1-Ig), PPARγ (Proteintech, China, 16643-1-Ig), CEBPα (Proteintech, Wuhan, China, 18311-1-AP), TUBB (Abclonal, Wuhan, China, AC021), ACTB (Proteintech, Wuhan, China, 66099-1-Ig), HRP-labeled Goat Anti-Rabbit IgG secondary antibody (Abclonal, Wuhan, China, AS003), HRP-labeled Goat Anti-Mouse IgG secondary antibody (Abclonal, Wuhan, China, AS014), ABflo^®^ 594-conjugated Goat Anti-Rabbit IgG (H + L) secondary antibody (Abclonal, Wuhan, China, AS003/AS014).

RNA Extraction Kit (Promega, Madison (Fitchburg), WI, USA, LS1040), cDNA Synthesis Kit (Novoprotein, Pudong, Shanghai, China, E042-01B), SYBR reagent (Novoprotein, Pudong, Shanghai, China, E401-01A), TAG Assay Kit (Beyotime, Songjiang, Shanghai, China, S0219S), BCA Protein Quantification Kit (Thermo Fisher Scientific, Grand Island, NY, USA, OB184645).

### 2.4. Isolation and Culture of Porcine Preadipocytes

After anesthesia and euthanasia, the pig was sterilized with 75% ethanol and transferred to a biosafety cabinet. Subcutaneous adipose tissue from the neck was collected. The tissue was rinsed once with 75% ethanol, then washed three times with PBS. The adipose tissue was transferred to a centrifuge tube and minced into pieces of approximately 1 mm^3^. A five-fold volume of 2mg mL^−1^ Type I collagenase was added, and digestion was carried out at 37 °C for 60 min. Digestion was terminated by adding an equal volume of DMEM complete medium supplemented with 10% FBS. The tissue digest was filtered through a 100 μm cell strainer and centrifuged at 1000 rpm for 8 min. The supernatant was discarded. Red blood cell lysis buffer was added, and lysis proceeded at room temperature for 10 min. Centrifugation was repeated at 1000 rpm for 8 min, and the supernatant was discarded. Finally, the cell pellet was resuspended in DMEM medium containing 20% FBS. The cells were seeded into culture dishes and incubated at 37 °C with 5% CO_2_. The complete medium was changed every 2 days. When cells reached 80% confluence, they were digested and passaged. Cells were seeded into 12-well plates at a density of 2 × 10^4^ cells per well. The day of seeding was designated as day 0. Cell counts were performed every 2 days to generate proliferation curves for the preadipocytes.

### 2.5. Immunofluorescence Identification of Beijing Black Pig Preadipocytes

Cells were seeded into 24-well plates. The next day, the medium was removed, and cells were washed three times with PBS; fixed with 4% PFA for 15 min, followed by three PBS washes; permeabilized with 0.1% Triton X-100 for 15 min, followed by three PBS washes; and blocked with 0.2% BSA for 60 min. After discarding the blocking solution, cells were incubated with DLK1 primary antibody (1:100 dilution) at 4 °C overnight, washed three times with PBS, incubated with fluorescent secondary antibody (1:100 dilution) at room temperature for 1 h, washed three times with PBS, and stained with 2 μg·mL^−1^ DAPI in the dark for 5 min, followed by three PBS washes. Cells were observed and photographed under a fluorescence inverted microscope.

### 2.6. Cell Proliferation Assay

Preadipocytes in the logarithmic growth phase were collected and seeded into 96-well plates at 4 × 10^3^ cells per well, followed by incubation at 37 °C with 5% CO_2_. After cell attachment, the original medium was replaced with one of the three adipogenic induction differentiation media. The control group received a basal medium containing amounts equivalent to those in the induction media, and a blank group contained no cells. Each group had 9 replicate wells. At days 0, 2, 4, 6, 8, and 10 after medium change, the absorbance at 450 nm was measured using the CCK-8 method. On the day of detection, the medium was replaced with serum-free medium containing 10% CCK-8 solution, and incubation continued for 3 h before measuring the OD450 value using a microplate reader. Using day 0 as the reference, the relative proliferation fold for each group was calculated as follows: Relative proliferation fold = (OD experimental group − OD blank group)/(OD control group − OD blank group).

### 2.7. EdU Staining

Following the instructions of the EdU detection kit, cells were treated with EdU. Subsequently, the culture medium was discarded, and cells were fixed with 4% paraformaldehyde. After rinsing, cells were permeabilized with 0.5% Triton X-100. Click reaction solution was added to cover the samples evenly, followed by incubation at room temperature in the dark for 30 min. Then, DAPI was added to counterstain the nuclei. Images were captured using a fluorescence microscope. Blue fluorescence represents the total number of detected cells, and red fluorescence represents proliferating cells. The percentage of EdU-positive cells (%) was calculated as (Number of red fluorescent cells/Number of blue fluorescent cells) × 100.

### 2.8. Adipogenic Induction and Differentiation of Beijing Black Pig Preadipocytes

After preadipocytes reached full confluence, they were cultured for an additional 2 days. Then, three different induction differentiation media (Table 1) were added, respectively, and maintained for 4 days, followed by replacement with maintenance differentiation media (Table 1) for another 4 days. The medium was changed every 2 days. The final concentrations of induction reagents in Table 1 were as follows: Insulin (INS) 5 μg·mL^−1^, Dexamethasone (DEX) 0.4 μg·mL^−1^, IBMX 0.5 mmol·L^−1^, Rosiglitazone (RSG) 1 μmol·L^−1^, Sodium Oleate (NaOL) 150 μmol·L^−1^, Biotin 33 μmol/L, Pantothenic Acid (PA) 17 μmol/L, Transferrin (TRF) 10 μg/mL.

### 2.9. Staining of Beijing Black Pig Adipocytes

The SFM-3 differentiation protocol was selected. Adipocytes from day 0 to day 8 of differentiation were subjected to Oil Red O staining and BODIPY staining, respectively. The medium was discarded, and cells were washed once with PBS, fixed with 4% PFA for 10 min, and washed twice with PBS. The subsequent procedures were as follows:

Oil Red O Staining: Incubate with staining wash solution for 20 s; discard the wash solution, add Oil Red O staining solution, and stain in the dark for 15 min; discard the Oil Red O staining solution, incubate with staining wash solution for 30 s; discard the wash solution, then add PBS, observe and photograph under a microscope

BODIPY Staining: Add staining solution containing 5 μmol·L^−1^ BODIPY and 2 μg·mL^−1^ DAPI to stain lipid droplets and nuclei in the dark for 10 min; discard the staining solution, wash three times with PBS, then observe and photograph using a fluorescence inverted microscope.

### 2.10. Measurement of Cellular TAG Content

A TAG assay kit was used to measure the TAG content in adipocytes induced to differentiate with SFM-3 from day 0 to day 8. Specific steps followed the kit instructions. The triglyceride content was normalized to the protein concentration per milligram.

### 2.11. Total RNA Extraction and cDNA Synthesis

Total RNA was extracted from cells induced with SFM-3 for 0, 2, 4, 6, and 8 days using the Promega RNA extraction kit. 1 μg of RNA was used for cDNA synthesis. The reaction system was: 2× NovoScript plus 1st Strand cDNA Synthesis SuperMix 10 μL, RNA 1 μg, gRNA Purge 1 μL, supplemented with RNase-free water to a total volume of 20 μL. The reaction program was: incubation at 50 °C for 15 min, followed by 85 °C for 5 s. After the reaction, the cDNA was diluted 10-fold and stored at −20 °C for later use.

### 2.12. Quantitative Real-Time PCR (qPCR)

Specific primers for qPCR were designed using the pick primer tool on the NCBI website (Table 2). 18S rRNA was used as the housekeeping gene. Real-time qPCR was performed using the Roche LightCycler 480 II. The reaction system was 20 μL: 2× Novo Start SYBR qPCR SuperMix Plus 10 μL, 0.5 μL each of forward and reverse primers (10 μmol·L^−1^), 2 μL template cDNA, and 7 μL ddH_2_O. The reaction conditions were: pre-denaturation at 95 °C for 1 min; then 40 cycles of 95 °C for 20 s, 60 °C for 20 s, and 72 °C for 30 s. The relative gene expression was calculated using the 2^−ΔΔCt^ method.

### 2.13. Total Protein Extraction and Western Blot

Cells after SFM-3 induction and differentiation were washed three times with PBS. RIPA lysis buffer was added, and cells were incubated on ice for 10 min. Cells were scraped, and the lysate was collected. The lysate was then centrifuged at 4 °C, 12,000 rpm for 30 min. The supernatant, containing the cellular protein, was collected. Proteins were denatured and loaded onto SDS-PAGE gels. After electrophoresis, proteins were transferred to PVDF membranes. Membranes were blocked with 5% skim milk at room temperature for 1 h, incubated with primary antibodies at 4 °C overnight, washed three times with TBST, incubated with secondary antibodies at room temperature for 40 min, and washed three times with TBST. ECL luminescent solution was added for color development, and the membranes were imaged and saved using a chemiluminescence instrument.

### 2.14. Cells and Transfections

Cells were maintained in complete DMEM medium supplemented with 10% fetal bovine serum (FBS; PAN-Seratech, Aidenbach, Germany) and 1% penicillin-streptomycin solution (PS; HyClone, Logan, UT, USA) under standard culture conditions (37 °C, 5% CO_2_). Plasmids for SEPTIN4 knockdownwere generated by Beijing Qingke Biotechnology Co., Ltd. (Beijing, China), and verified by DNA sequencing. Cells were infected with the SEPTIN4 knockdown palsmids and selected with puromycin according to the manufacturer’s instructions.

### 2.15. Statistical Analysis

Statistical analysis was performed using GraphPad Prism 8.0 (GraphPad Prism Software). All values are expressed as mean ± SD. For comparisons between two groups, a *t*-test was used after assessing normality. For comparisons among more than two groups, one-way analysis of variance (ANOVA) followed by Tukey’s test for multiple comparisons was used. *p* < 0.05 was considered statistically significant (* *p* < 0.05, ** *p* < 0.01, *** *p* < 0.001).

## 3. Results

### 3.1. Isolation, Culture, and Identification of Porcine Preadipocytes

**Figure 1:**
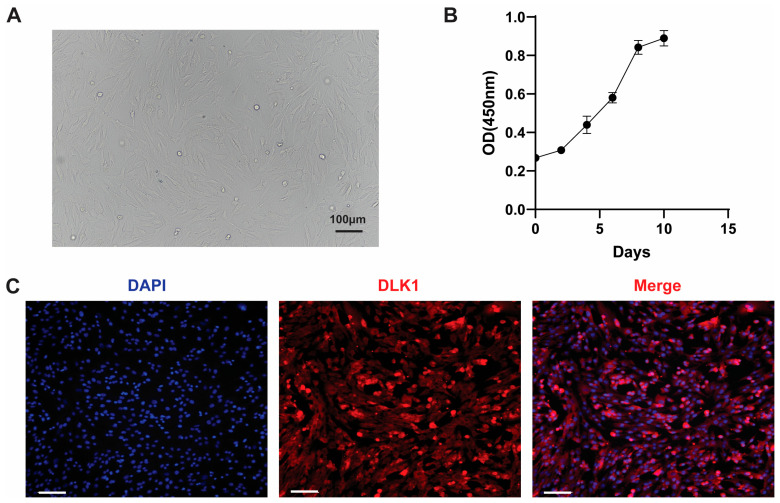
Isolation and characterization of porcine preadipocytes. (**A**) Phase-contrast images showing primary porcine preadipocytes 24 h after isolation exhibiting spindle-shaped morphology. (**B**) Proliferation curve of preadipocytes cultured in growth medium, demonstrating a characteristic lag, exponential, and plateau phase. (**C**) Immunofluorescence staining confirming DLK1 expression in primary preadipocytes. Nuclei were counterstained with DAPI. Blue: DAPI, Red: DLK1, Scale bars: 100 μm.

Obtaining highly purified preadipocytes is a prerequisite for in vitro adipogenesis research. Consequently, primary cells were successfully isolated from the neck subcutaneous adipose tissue of Beijing Black pigs using a combination of mechanical method and Type I collagenase digestion. Within 24 h post-seeding, most cells adhered, exhibiting an irregular elongated spindle morphology under the microscope (Figure 1A). After passaging, the growth curve plotted using the CCK-8 assay showed a typical “S”-shaped growth trend: a lag phase from days 0 to 4, a logarithmic growth phase with rapid proliferation from days 4 to 8, followed by entry into the plateau phase around day 8 due to contact inhibition (Figure 1B), consistent with the general pattern of in vitro cell culture. To further identify the cell type, immunofluorescence staining was performed on passaged cells. As shown in Figure 1C, the detection of DLK1, a specific marker protein for preadipocytes, yielded positive results, indicating high purity of the isolated cell population. This study successfully established the isolation and culture system for preadipocytes from Beijing Black pigs. The obtained cells exhibited typical precursor cell morphology and growth characteristics and were confirmed by DLK1 protein identification, providing reliable experimental material for subsequent serum-free adipogenic induction and differentiation studies.

### 3.2. Effect of SFM-1 on Adipogenic Differentiation of Porcine Preadipocytes

**Figure 2:**
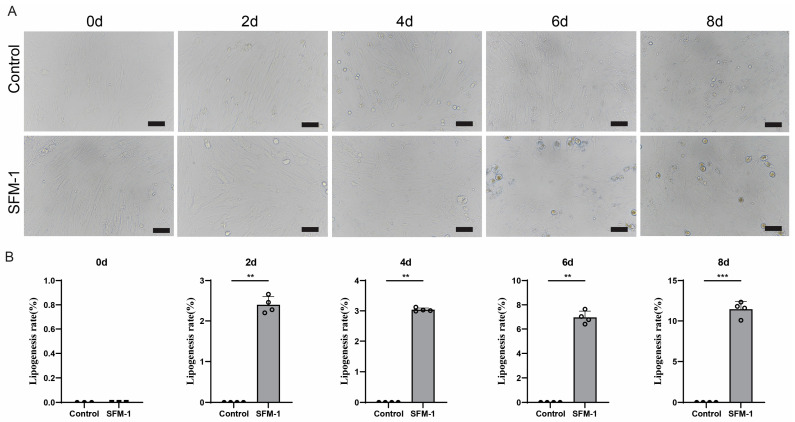
Serum-free basal induction medium (SFM-1) initiates adipogenic differentiation. (**A**) Representative micrographs of cells induced with SFM-1 from day 0 to day 8, showing gradual intracellular lipid deposition. (**B**) Oil red O staining quantification of lipid accumulation demonstrating low differentiation efficiency under SFM-1 conditions. Scale bars: 50 μm. Data are mean ± SD; ** *p* < 0.01, *** *p* < 0.001.

To establish a serum-free adipogenic induction system, it was first necessary to verify the feasibility of the basic induction formula. This section aimed to evaluate the ability of the basic serum-free differentiation medium 1 (SFM-1) to initiate the adipogenic differentiation of porcine Preadipocytes. After induction with the SFM-1 protocol, a small number of lipid droplets began to appear in the cells from day 2. The number of lipid droplets showed a slow increasing trend with prolonged induction time, and obvious lipid droplet accumulation could be observed by day 8 (Figure 2A). However, compared to the typical lipid droplet morphology and abundance of mature adipocytes, the SFM-1 group cells had relatively fewer and smaller lipid droplets overall. Oil red O staining quantitative analysis confirmed a low level of differentiation efficiency, reaching only about 10% by day 8 (Figure 2B). Although the basal serum-free medium (SFM-1) initiated the differentiation program and promoted intracellular lipid deposition, its induction efficiency was limited and insufficient for robust adipogenic differentiation. These results indicate that the basal formula alone is insufficient for efficient induction. This suggests that adding key auxiliary factors in subsequent formulations could synergistically enhance lipid synthesis and accumulation, guiding further system optimization.

### 3.3. Effect of SFM-2 on Adipogenic Differentiation of Porcine Preadipocytes

**Figure 3:**
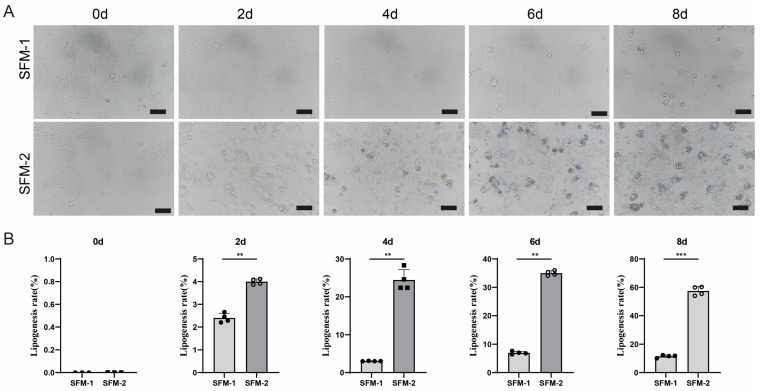
Oleic acid supplementation (SFM-2) enhances lipid accumulation. (**A**) Time-course images showing increased intracellular lipid deposition under SFM-2 compared with SFM-1. (**B**) Oil red O staining quantification analysis of intracellular lipid content demonstrating significantly greater accumulation in SFM-2. Scale bars: 50 μm. Data are mean ± SD; ** *p* < 0.01, *** *p* < 0.001.

Addressing the low differentiation efficiency of the SFM-1 protocol, the study investigated signal activation and transduction processes in in vivo fat synthesis, where oleic acid (OA) might synergistically promote differentiation by providing lipid synthesis substrates and activating key signaling pathways. Therefore, this study added OA to the basic induction medium to form serum-free differentiation medium 2 (SFM-2) and investigated its effect on the adipogenic differentiation of porcine Preadipocytes. Compared to the SFM-1 protocol, lipid droplets were observed in cells induced with SFM-2 as early as day 2, and the number and volume of lipid droplets continuously increased during the induction process from days 4 to 8, with the difference being significantly higher than SFM-1 (Figure 3A). Oil red O staining quantitative analysis results showed that the lipid accumulation level in the SFM-2 group was significantly higher than in the SFM-1 group, indicating a significant improvement in adipogenic differentiation efficiency (Figure 3B). Adding OA to the basic induction medium significantly promoted intracellular lipid deposition and lipid accumulation in porcine Preadipocytes, demonstrating that OA is a key factor driving adipocyte differentiation and maturation in the serum-free system. These findings support the hypothesis that OA enhances adipogenesis through both metabolic and signaling pathways. These results clarified the important role of OA in serum-free adipogenic induction, providing a key basis and optimization direction for constructing a more complete and efficient serum-free differentiation system.

### 3.4. Effect of SFM-3 on Adipogenic Differentiation of Porcine Preadipocytes

**Figure 4:**
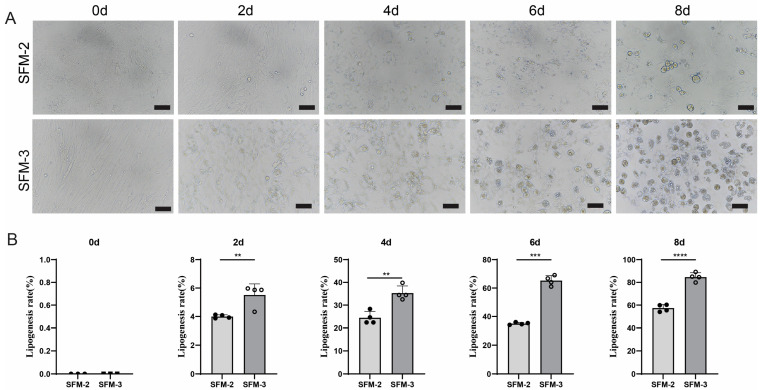
Transferrin addition promotes robust adipocyte maturation. (**A**) Representative images of cells induced with SFM-3, showing extensive intracellular lipid deposition by day 8. Scale bars: 50 μm. (**B**) Oil red O staining quantification confirming significantly higher lipid accumulation in SFM-3 versus SFM-1 and SFM-2. Scale bars: 50 μm. Data are mean ± SD; ** *p* < 0.01, *** *p* < 0.001, **** *p* < 0.0001.

**Figure 5:**
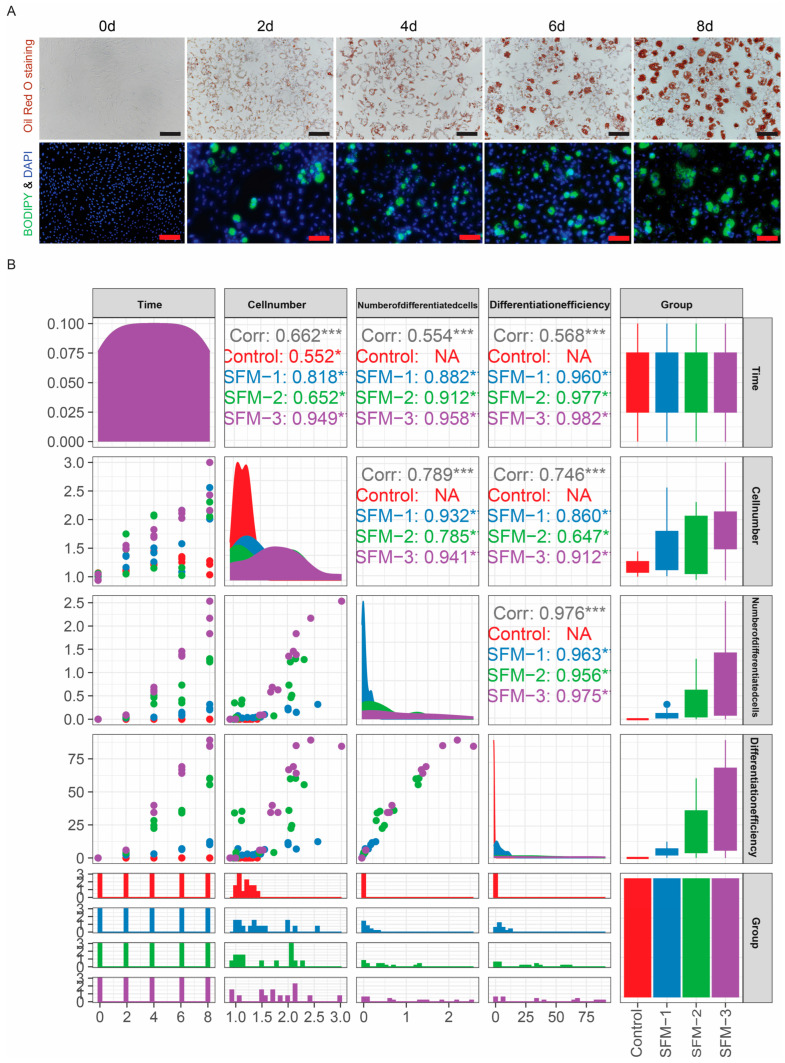
Fluorescent lipid staining and correlation analysis of differentiation efficiency. (**A**) Oil Red O and BODIPY staining of cells treated with SFM-3 from day 0 to day 8 showing progressive lipid accumulation. Scale bars: 50 μm. Red: Oil Red O; Green: BODIPY; Blue: nuclei. (**B**) Correlation analysis demonstrating strong association between treatment condition and lipid deposition efficiency. * *p* < 0.05, *** *p* < 0.001.

To further achieve efficient adipogenesis suitable for scalable applications, and based on the proven differentiation-promoting effect of the SFM-2, this study further explored other signaling pathways and key promoting factors that might have synergistic effects, hypothesizing that transferrin (TRF) might have a synergistic efficacy-enhancing role. By constructing a complete serum-free differentiation medium 3 (SFM-3) with added OA and TRF, the aim was to achieve efficient and complete differentiation of porcine Preadipocytes to meet the demand for fat component generation efficiency in the industrialization of cultured meat. The research results showed that the SFM-3 protocol exhibited markedly enhanced adipogenic induction. Lipid droplets appeared in cells as early as day 2 of induction, their number and volume continuously and significantly increased during days 4–6, and reached the peak of differentiation by day 8, with cells filled with abundant lipid droplets (Figure 4A). Oil red O staining and quantitative analysis results indicated that the differentiation efficiency of SFM-3 was significantly better than that of the SFM-1 and SFM-2 protocols (Figure 4B and Figure 5A), indicating that the introduction of TRF further enhanced the differentiation process. These results indicate that SFM-3, constructed by supplementing SFM-2 with TRF, can induce porcine preadipocytes to undergo adipogenic differentiation more rapidly and efficiently, yielding functionally mature adipocytes. This regimen demonstrated the highest efficacy in our study. The establishment of the SFM-3 represents a significant advance in serum-free induction technology for porcine preadipocytes. It not only provides a key technical solution to the bottleneck of insufficient adipogenic differentiation efficiency in the industrialization of cultured meat but also offers a more ideal in vitro model for in-depth study of the mechanisms of adipocyte differentiation.

### 3.5. Staining of Porcine Adipocytes and Correlation Analysis Between Experimental Groups and Target Variables

Having established the optimal induction effect of the SFM-3, we further verified the biological characteristics of the lipid droplets it induced and systematically evaluated the quantitative relationship between different treatment conditions and adipogenic differentiation efficiency. Oil Red O and BODIPY staining of cells induced with SFM-3 for 0–8 days showed no lipid droplet staining on differentiation day 0; from day 2 onwards, red (Oil Red O) and green (BODIPY) fluorescent signals appeared, and the signal intensity continuously increased over time, peaking at day 8, indicating massive intracellular lipid deposition (Figure 5A). Correlation analysis further revealed that all treatment groups showed a significant positive correlation with cell differentiation efficiency, with the SFM-3 treatment group having the strongest correlation (correlation coefficient r = 0.982, *p* < 0.05), significantly higher than the SFM-1 (r = 0.960) and SFM-2 (r = 0.977) groups (Figure 5B). The staining results confirmed that the SFM-3 can effectively induce preadipocytes to synthesize and accumulate neutral lipids, forming functionally mature adipocytes. The correlation analysis statistically confirmed the significant advantage of SFM-3 in promoting adipogenic differentiation. These results validate the high efficiency and reliability of the SFM-3 from both morphological and statistical perspectives. This provides a solid basis for its application as a standardized serum-free adipogenic induction protocol and advances the technical framework for controlled production of fat components in cultured meat.

### 3.6. Effects of Three Serum-Free Differentiation Regimens on the Proliferation of Porcine Preadipocytes

**Figure 6:**
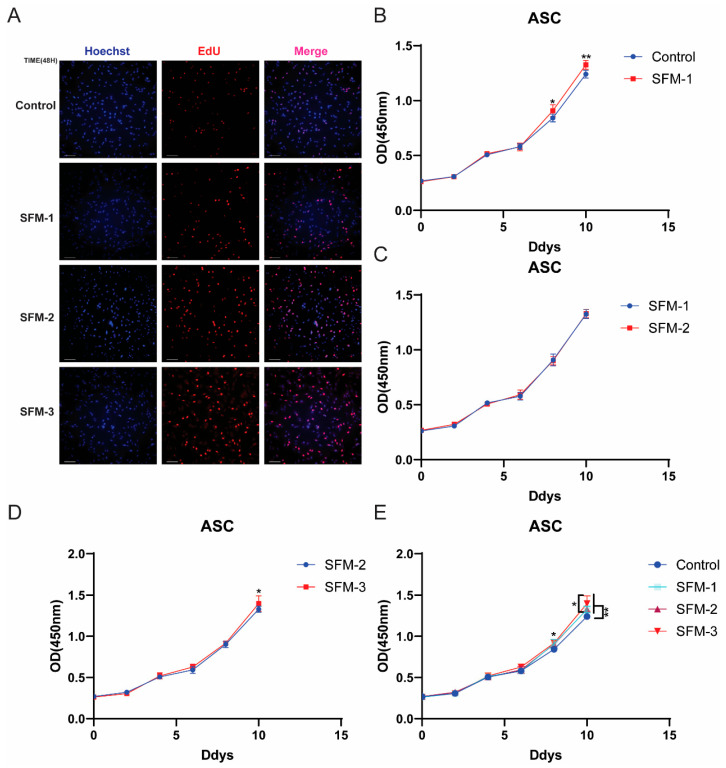
Effects of serum-free regimens on preadipocyte proliferation. (**A**) EdU staining showing increased proliferative cells under SFM treatments relative to control. Blue: Hoechst, Red: EdU. (**B**–**E**) CCK-8 assay quantifying proliferation dynamics over 10 days for SFM-1, SFM-2, and SFM-3 groups. Data are mean ± SD; * *p* < 0.05, ** *p* < 0.01 versus control.

Induction media that promote differentiation may sometimes inhibit or antagonize cell proliferation. To clarify the appropriate addition timing of the induction media used in this study and confirm the impact of differentiation conditions on cell proliferation, and based on the potential roles of OA and TRF in cell metabolism, the study first evaluated the effects of three serum-free differentiation regimens (SFM-1, SFM-2, SFM-3) on the proliferation capacity of preadipocytes using EdU staining and CCK-8 assay to determine the optimal pretreatment conditions. EdU staining showed that after 48 h of treatment with the three regimens, the number of cells in the proliferative state was significantly higher than in the control group (Figure 6A). The EdU-positive cell rate in the SFM-2 and SFM-3 groups was significantly higher than in the SFM-1 group (*p* < 0.05), while there was no significant difference between the SFM-2 and SFM-3 groups. The CCK-8 assay results monitored continuously for 10 days further indicated: SFM-1/2 treatments began to show pro-proliferative effects by day 8, reaching significant levels by day 10 (*p* < 0.01); SFM-3 maintained the highest pro-proliferative capacity throughout the treatment period, being significantly better than SFM-1 and SFM-2 by day 10 (*p* < 0.05) (Figure 6B–E). The above results indicate that all three serum-free differentiation regimens can promote the proliferation of porcine Preadipocytes, with SFM-3 (containing OA and TRF) exhibiting the strongest pro-proliferative effect, suggesting a synergistic effect of OA and TRF in maintaining precursor cell viability. Although the core objective of this study was to establish a serum-free adipogenic differentiation system, cell proliferation and differentiation are closely linked continuous processes—sufficient numbers of precursor cells are the foundation for efficient differentiation. The SFM-3 protocol supported both proliferation and subsequent differentiation, while also confirming that the protocols in this study do not inhibit cell proliferation. This experiment confirmed the combined advantages of the SFM-3 protocol in promoting cell expansion and differentiation, which provided a necessary prerequisite for its subsequent excellent differentiation performance, further illustrating the value of this system in the entire in vitro fat generation process.

### 3.7. Investigation of the Mechanism of SFM-3-Induced Adipocyte Differentiation

**Figure 7:**
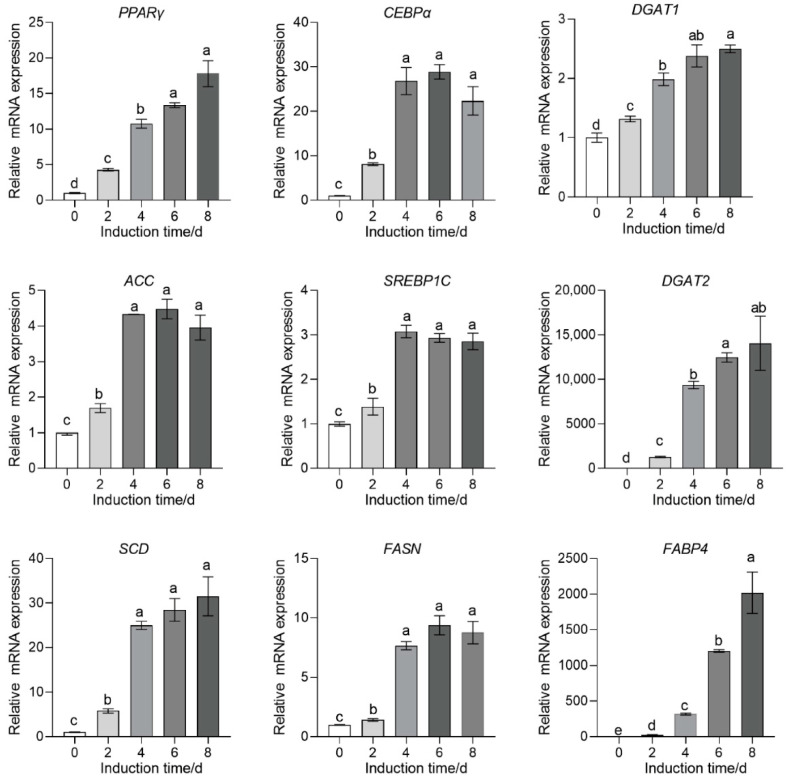
Temporal activation of adipogenic gene expression under SFM-3 induction. Relative mRNA expression of key transcription factors and lipid-metabolic genes during adipogenic differentiation (days 0–8). PPARγ and CEBPα activated early, followed by lipogenic and maturation markers. Different lowercase letters indicate significant differences (*p* < 0.05), while the same lowercase letters represent no significant differences (*p* > 0.05).

Morphological observations and staining results confirm the potent adipogenic capacity of SFM-3. To further explore its regulatory mechanisms at the molecular level, this study systematically analyzed the expression of multiple key adipogenic genes under SFM-3 induction using qPCR. The results showed that in the early stage of differentiation (day 2), the core transcriptional regulators PPARγ and CEBPα were significantly upregulated (*p* < 0.05), while fatty acid synthesis-related genes (ACC, SREBP-1c, FASN) and DGAT1 responded rapidly. During days 4–6, the expression of PPARγ, CEBPα, ACC, DGAT1, SREBP-1c, and FASN peaked, dominating the lipid synthesis stage. By the late differentiation stage (day 8), the focus of gene expression shifted towards lipid assembly and functional maturation, with DGAT2, SCD, and the mature adipocyte marker FABP4 reaching their highest expression levels (Figure 7), exhibiting a highly coordinated temporal regulation pattern. These results indicate that the SFM-3 activated the complete adipogenic gene regulatory network. The temporal expression pattern closely mirrored that of in vivo adipose development, confirming at the molecular level that this system drives preadipocytes through the entire differentiation process, from initiation to functional maturation. Detecting gene expression during differentiation not only verified the high efficiency of the SFM-3 at the transcriptional level but, more importantly, mapped the molecular landscape of porcine adipocyte differentiation under serum-free conditions, providing precise molecular targets for optimizing the induction protocol and establishing a reliable molecular marker system for assessing the functional maturity of adipose tissue in cultured meat.

### 3.8. Expression of Related Proteins and TAG Content Measurement During Adipocyte Differentiation

**Figure 8:**
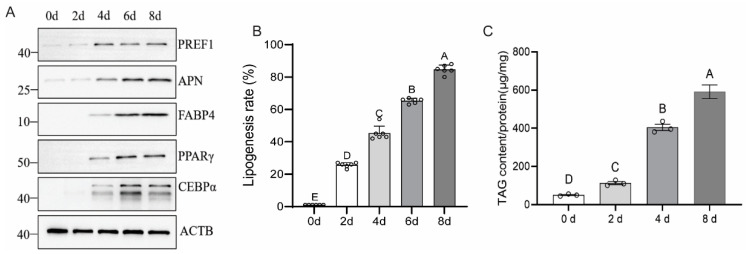
Protein expression and triglyceride accumulation during serum-free adipogenesis. (**A**) Western blot showing progressive activation of PPARγ, CEBPα, APN, and FABP4, and reduction in PREF1. (**B**,**C**) Quantification of differentiation efficiency and triglyceride content across differentiation timeline. Different capital letters indicate significant differences (*p* < 0.05), while the same capital letters represent no significant differences (*p* > 0.05).

Gene expression analysis has confirmed the effectiveness of the SFM-3 at the transcriptional level. However, as the direct executors of function, protein expression dynamics better reflect the true state of the cells. This study detected the expression changes in key proteins during differentiation using Western blot technology, combined with triglyceride (TAG) content measurement, to evaluate the maturity of adipocytes at the functional level. Western blot results showed that the core transcription factors PPARγ and CEBPα were continuously upregulated during differentiation, consistent with the gene expression trend. The expression of adipocyte functional proteins APN and FABP4 significantly increased with the differentiation process, indicating that the cells acquired mature metabolic functions (Figure 8A, Supplementary Figure S1). The precursor cell marker PREF1 showed a transient increase in the early stage of differentiation, peaked at day 4, and then significantly decreased, confirming that the cells had successfully exited the precursor state (Figure 8A, Supplementary Figure S1). TAG content measurement showed that starting from day 2 of differentiation, the intracellular TAG content continuously increased over time, peaking at day 8 (*p* < 0.001), which was fully consistent with the lipid droplet accumulation observed morphologically (Figure 8B,C). Protein-level analysis confirmed that the SFM-3 can effectively drive the functional maturation of adipocytes, manifested as the continuous activation of core transcription factors, significant expression of functional proteins, and timely silencing of the precursor marker. The dynamic changes in TAG content further verified the cells’ lipid synthesis and storage capacity. Furthermore, the detection of protein expression during differentiation compensated for the limitations of transcriptional level analysis, providing direct evidence that the SFM-3 induces the generation of functionally mature adipocytes. The combination of TAG content and protein expression provided integrated evidence across molecular and functional levels from molecular regulation to functional performance, providing a key indicator system for quality assessment of fat components in cultured meat.

### 3.9. High Expression of SEPTIN4 Is Significantly Associated with SFM-3-Induced Adipocyte Differentiation

**Figure 9:**
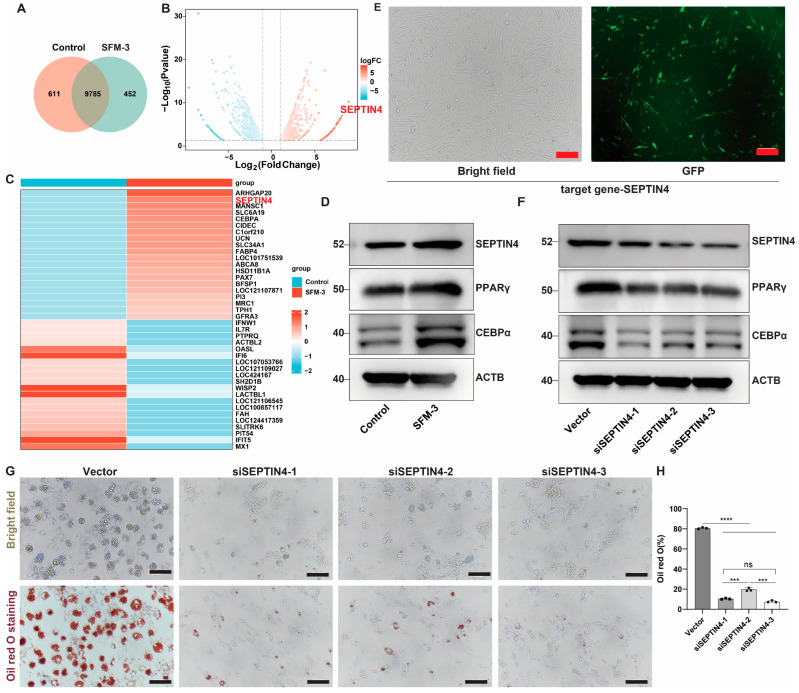
High expression of SEPTIN4 is highly correlated with adipogenesis. (**A**,**B**) Venn diagram and Volcano plot of differentially expressed proteins between the SFM-3 group and the control group, with a fold change ≥ 1.5 and adjusted *p* < 0.01. (**C**) The analysis of differential genes was enriched, with SEPTIN4 ranking second in the small molecule metabolic process. (**D**) Western blotting of SEPTIN4, PPARγ and CEBPα in SFM-3 induced or non-induced groups. (**E**) Representative images of adipocytes down-expressing SEPTIN4. Scale bars, 100 µm. (**F**) Western blotting verified the protein levels of SEPTIN4, PPARγ and CEBPα in adipocytes. (**G**) Representative bright field and oil red O staining images of cells induced with SFM-3 in 4 groups (Vector, siSEPTIN4-1, siSEPTIN4-2, siSEPTIN4-3), Scale bars, 200 µm. (**H**) Assessment of oil red O (%) each group treated with SFM-3. Data are mean ± SD; *** *p* < 0.001, **** *p* < 0.0001, ns, not significant.

To further explore the specific mechanisms underlying the gene regulatory network following SFM-3-induced differentiation of porcine adipocyte precursors, we performed transcriptomic sequencing on adipocytes with or without SFM-3 induction for 8 days. A total of 10,848 genes were detected, of which 1063 were significantly differentially expressed (Figure 9A). A volcano plot illustrating the distribution of these genes is shown in Figure 9B. Based on previous studies, among the top 20 differentially expressed molecules, we focused on SEPTIN4, which ranked second, to validate its association with adipogenesis. Western blot analysis of adipocytes with or without SFM-3 induction for 8 days demonstrated that SEPTIN4 expression was significantly higher in the induced group compared to the control group (Figure 9C,D). To investigate whether SEPTIN4 drives adipocyte precursor differentiation, we knocked down SEPTIN4 in adipocytes using plasmid infection (Figure 9E). After confirming transfection efficiency by Western blot (Figure 9F), cells were induced with SFM-3 for 8 days. Results showed that the number and fusion of lipid droplets were markedly reduced in the SEPTIN4 knockdown group compared to the control group (Figure 9H). These results confirmed that high SEPTIN4 expression is positively correlated with SFM-3-induced adipocytes differentiation.

### 3.10. SEPTIN4 Induces Adipocyte Precursor Differentiation by Activating the PPARγ Signaling Pathway

**Figure 10:**
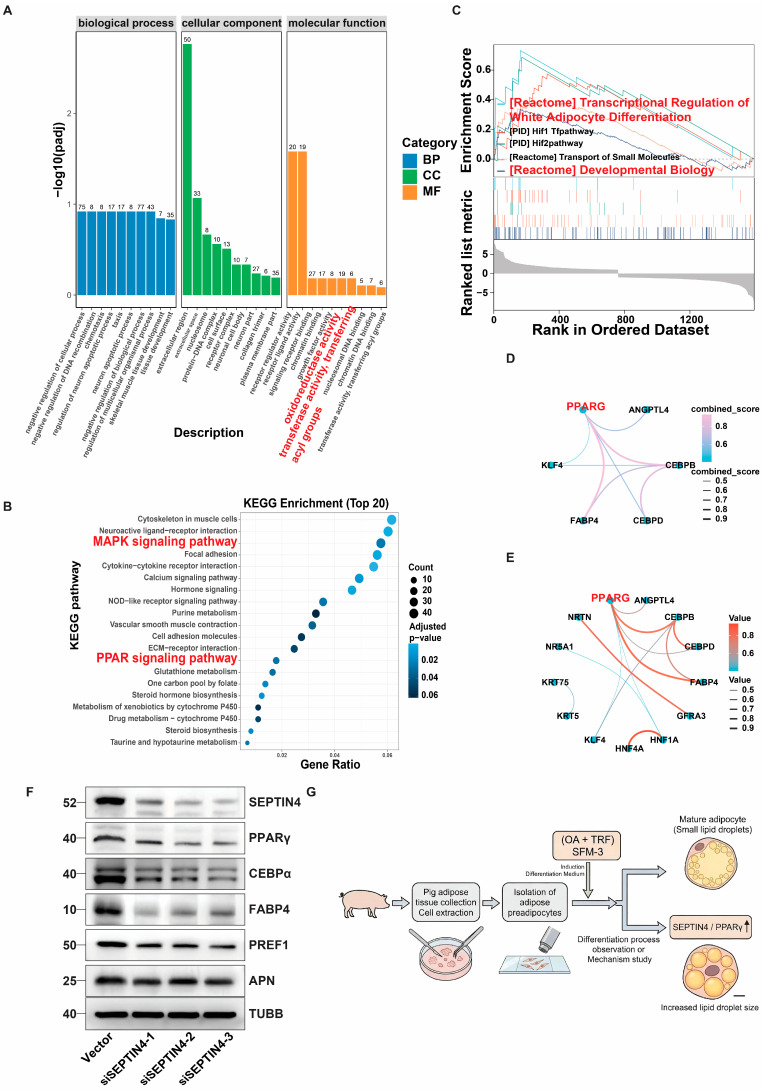
SEPTIN4 induces adipocyte precursor differentiation by regulating the PPARγ signaling pathway. (**A**) GO enrichment analysis of differentially expressed genes between adipocytes with or without SFM-3 induction for 8 days. Genes associated with SFM-3-induced differentiation were mainly enriched in transferase activity (transferring acyl groups) and oxidoreductase activity. (**B**) KEGG pathway enrichment analysis of differentially expressed genes between the SFM-3-induced group and the control group, revealing activation of the PPARγ signaling pathway and MAPK signaling pathway. (**C**) GSEA showing that genes associated with SFM-3-induced adipocyte differentiation were mainly enriched in metabolite-related pathways, particularly [Reactome] Transcriptional Regulation of White Adipocyte Differentiation and [Reactome] Developmental Biology. (**D**,**E**) Enrichment plots from GSEA demonstrating that PPARγ occupies a central position in the [Reactome] Transcriptional Regulation of White Adipocyte Differentiation pathway (**D**) and the [Reactome] Developmental Biology pathway (**E**). (**F**) Western blot analysis of PPARγ, CEBPα, FABP4, PREF1, and APN protein levels in adipocytes with or without SEPTIN4 knockdown following 8 days of SFM-3 induction (related to Figure 9F and Figure 10F). SEPTIN4 knockdown suppressed the expression of these adipogenic markers. (**G**) Schematic diagram illustrating the experimental workflow and proposed molecular mechanism of SFM-3-induced adipogenic differentiation. Primary porcine preadipocytes were isolated from subcutaneous adipose tissue, identified by DLK1 immunofluorescence, and induced to differentiate using the serum-free SFM-3 system. Morphological evaluation by Oil Red O and BODIPY staining showed progressive lipid accumulation. Mechanistically, OA and TRF synergistically upregulate SEPTIN4, which activates the PPARγ signaling pathway, leading to increased expression of adipogenic markers (PPARγ, CEBPα, FABP4, APN) and decreased expression of the precursor marker PREF1, ultimately promoting adipocyte differentiation and functional maturation. The arrows indicate the experimental process. Data are mean ± SD.

GO enrichment analysis revealed that genes associated with SFM-3-induced adipocyte differentiation were related to transferase activity (transferring acyl groups) and oxidoreductase activity (Figure 10A). Enrichment of the KEGG signaling pathway revealed activation of the PPARγ signaling pathway associated with adipogenic factors and MAPK signaling pathway associated with cell proliferation (Figure 10B). GSEA further demonstrated that genes associated with SFM-3-induced adipocyte differentiation were mainly enriched in metabolite-related pathways, particularly [Reactome] Transcriptional Regulation of White Adipocyte Differentiation and [Reactome] Developmental Biology (Figure 10C), with PPARγ occupying a central position in both pathways (Figure 10D,E). To investigate the regulatory effect of SEPTIN4 on PPARγ, Western blot analysis was performed on adipocytes with or without SFM-3 induction for 8 days. Results showed that expression levels of both SEPTIN4, PPARγ and CEBPα were significantly higher in the induced group compared to the control group (Figure 9D). Western blot analysis of adipocytes with or without SEPTIN4 knockdown following 8 days of induction demonstrated that SEPTIN4 knockdown suppressed the expression of PPARγ, CEBPα, FABP4, PREF1, and APN (Figure 9F and Figure 10F,G). These results demonstrated that SEPTIN4 induces adipocyte precursor differentiation by regulating the PPARγ signaling pathway. To further summarize the overall experimental workflow and the proposed molecular mechanism, a schematic diagram was constructed. As illustrated in Figure 10G, primary porcine preadipocytes were isolated from subcutaneous adipose tissue, identified by DLK1 immunofluorescence, and subsequently induced to differentiate using the serum-free SFM-3 induction system. Morphological assessment via Oil Red O and BODIPY staining confirmed progressive lipid droplet accumulation, while mechanistic investigation revealed that the synergistic action of oleic acid (OA) and transferrin (TRF) in SFM-3 upregulates SEPTIN4 expression. SEPTIN4 then activates the PPARγ signaling pathway, leading to the upregulation of downstream adipogenic markers including CEBPα, FABP4, and APN, and the downregulation of the preadipocyte marker PREF1, thereby driving adipogenic differentiation.

## 4. Discussion

In the field of cultured meat research, although significant progress has been made in the directed differentiation of muscle tissue and the development of edible scaffolds [42], this technology still faces the major challenge of matching the taste and flavor of traditional meat products [34,43]. Fat content is a critical determinant of meat quality [16]. Therefore, the in vitro construction of adipose tissue has become a key focus in enhancing the sensory quality of the product. However, the efficient and defined adipose generation of fat components still faces key technical bottlenecks. Serum-dependent culture systems commonly used in current research have inherent limitations. The undefined composition of serum leads to unstable differentiation efficiency, while batch-to-batch variation compromises experimental reproducibility. These factors collectively impede the standardization of cultured fat production. Therefore, establishing a chemically defined, serum-free differentiation medium 3 with stable differentiation efficiency and no risk of exogenous contamination has become a critical issue to be solved for advancing cultured meat from the laboratory to industrialization [44]. Building upon established serum-free culture principles, this study aimed to develop an efficient and stable serum-free induction protocol for porcine preadipocytes through systematic optimization of induction components, providing a reliable technical platform for the standardized production of adipose tissue in cultured meat.

In the construction of the serum-free differentiation medium 3, oleic acid (OA) exhibited multi-level regulatory functions. The results of this study indicate that OA not only acts as a precursor for lipid synthesis [45,46] but also significantly promotes adipogenesis by activating the PPARγ signaling pathway [25,47]. This finding aligns with the results of M.C.R. et al., who demonstrated that OA promotes preadipocyte proliferation via the AKT2 signaling pathway [48]. Collectively, these studies reveal the multifaceted regulatory roles of OA in adipocyte development. Notably, this study found a significant synergistic effect between OA and transferrin (TRF) in the induction system: OA provides lipid synthesis substrates and activates key transcription factors, while TRF ensures the normal progress of metabolic processes by maintaining cellular iron homeostasis. The combination of OA and TRF exhibited a synergistic effect, exceeding what would be expected from an additive model in promoting adipocyte differentiation [49]. This synergistic mechanism has not been well characterized in previous studies and thus provides a new theoretical basis for optimizing serum-free culture systems.

TRF also regulates adipocyte differentiation. Our results demonstrate that the addition of TRF significantly improved the differentiation efficiency and functional maturity of preadipocytes. This finding is highly consistent with the results of Wang et al., where TRF influenced adipocyte fate by regulating iron homeostasis [50]. Further mechanistic studies indicate that TRF not only maintains cellular iron metabolic balance, ensuring the normal operation of enzyme systems related to lipid synthesis, but also coordinates the adipogenic differentiation process at the molecular level by promoting the activation of the PPARγ/CEBPα transcriptional network [50]. It is particularly noteworthy that TRF may influence the cell’s energy metabolism status by regulating mitochondrial function, providing necessary energy support for lipid synthesis [41,51]. This non-canonical regulatory function expands our understanding of TRF’s role in cellular metabolism and provides novel insights for the design of serum-free culture systems.

In this study, we elucidated the synergistic role of oleic acid (OA) and transferrin (TRF) in promoting adipogenesis through transcriptomic analysis, confirming their cooperative activation of the SEPTIN4/PPARγ/CEBPα axis. However, several limitations remain. For instance, the potential interplay between OA, a natural ligand of PPARγ, and the iron homeostasis maintained by TRF, as well as how this interaction precisely regulates the adipogenic differentiation process, warrants further investigation. Furthermore, whether OA exerts cross-regulation on TRF-mediated iron transport or signaling, and how this regulatory network fine-tunes the sequential activation of adipogenic genes, requires in-depth exploration. Additionally, the optimal ratio of OA to TRF and their dynamic regulatory patterns at different differentiation stages merit further study. Addressing these questions is essential for further refining the serum-free induction system and enhancing both the differentiation efficiency and functional maturation of adipocytes.

By systematically analyzing the dynamics of gene and protein expression during differentiation, this study successfully mapped the molecular landscape of adipocyte differentiation under serum-free induction conditions. The study showed that PPARγ/CEBPα were significantly upregulated in the early stage of differentiation, driving the cascade activation of the downstream lipid synthesis gene network. This temporal characteristic highly coincided with the in vivo adipose development process [23,24]. Notably, the expression dynamics of the precursor cell marker PREF1 provided a reliable molecular marker for assessing the differentiation progress [52,53]. While the classic pattern in murine and human adipogenesis is a steady decline of the preadipocyte marker PREF1, our observation of a transient peak at day 4 in porcine cells aligns with findings in other species-specific differentiation models. This may reflect a distinct regulatory dynamic in porcine adipocytes, where an initial feedback loop transiently upregulates PREF1 to regulate the timing of exit from the precursor state before terminal differentiation commences, potentially synchronizing the process. This finding provides a new benchmark for evaluating the functional maturity of adipocytes in serum-free culture systems. This molecular map not only reveals the dynamic regulatory mechanism of adipocyte differentiation but also provides precise target references for optimizing the induction protocol.

Although this study successfully established a highly efficient SFM-3, a direct comparison with serum-containing induction media was not performed. Future work will be essential to benchmark the differentiation efficiency and functional maturity of SFM-3-induced adipocytes against current gold-standard serum-based systems. Nevertheless, the achieved differentiation efficiency and the clear activation of the adipogenic molecular network demonstrate that SFM-3 represents a robust, chemically defined alternative that overcomes the critical drawbacks associated with serum use.

Collectively, this study represents a significant breakthrough in adipose tissue construction technology for cultured meat by establishing an efficient serum-free induction system for porcine adipocyte precursors. At the technical level, we developed a chemically defined culture system that ensures stable differentiation efficiency for in vitro adipogenesis. At the mechanistic level, we elucidated the molecular regulatory network by which OA and TRF synergistically modulate the SEPTIN4/PPARγ/CEBPα axis. At the application level, this work provides a reliable cell culture protocol for the industrialization of cultured meat. By effectively resolving the serum dependency bottleneck in adipose tissue engineering, this study lays a solid foundation for improving the flavor quality and advancing the standardized production of cultured meat, holding significant strategic importance for promoting the development of the cellular agriculture industry.

## 5. Conclusions

This study demonstrated that the establishment of a serum-free isolation and efficient adipogenic differentiation system for porcine adipocyte precursors, through the SFM-3 induction protocol, represents a pivotal advance for cultured meat production. We delineate a mechanism whereby the synergistic action of oleic acid and transferrin orchestrates a coordinated adipogenic program: it activates the SEPTIN4/PPARγ/CEBPα core regulatory network, which in turn drives the sequential expression of adipogenic genes and fuels substantial lipid droplet accumulation, culminating in the functional maturation of adipocytes within 8 days. This system thus concurrently overcomes the serum dependency bottleneck and ensures stable differentiation capacity. The reversal of serum dependency by this chemically defined formulation underscores the vulnerability created by conventional culture paradigms. Our study not only elucidates a previously unrecognized interplay between lipid metabolism regulators (oleic acid/transferrin) and the adipogenic transcriptional cascade in driving efficient adipogenesis but also nominates this serum-free platform as a promising strategy for the standardized production of adipose tissue components, offering significant value for improving the flavor quality of cultured meat and advancing industrial standardization.

## Figures and Tables

**Table 1:** Induction and maintenance differentiation media.

Group	Induction Differentiation Media	Maintenance Differentiation Media
SFM-1	DMEM + INS + Biotin + PA + DEX + IBMX + RSG	DMEM + INS + Biotin + PA + DEX
SFM-2	DMEM + INS + Biotin + PA + DEX + IBMX + RSG + OA	DMEM + INS + Biotin + PA + DEX + OA
SFM-3	DMEM + INS + Biotin + PA + DEX + IBMX + RSG + OA + TRF	DMEM + INS + Biotin + PA + DEX + OA + TRF

**Table 2:** Primer sequences used for real-time quantitative PCR.

Gene	Accession Numbers	Sequence (5′ ⟶ 3′)	Product Length	Tm	GC%
*PPARγ*	NM_214379.1	F: CCAGCATTTCCACTCCACACTA R: GACACAGGCTCCACTTTGATG	124	F: 60.29 R: 59.19	F: 50.00 R: 52.83
*CEBPα*	XM_003127015.4	F: AGCCAAGAAGTCGGTAGA R: CGGTCATTGTCACTGGTC	150	F: 54.73 R: 55.40	F: 50.00 R: 55.56
*DGAT1*	XM_021088697.1	F: CCCACCATCCAGAACTCCAT R: CGGTCTCCAAACTGCATGAG	170	F: 59.08 R: 58.92	F: 55.00 R: 55.00
*ACC*	NM_001114269.1	F: TCAGAAGGAGGAGGAGGGAA R: ATGACGGGACTGTTTGGCTA	135	F: 58.91 R: 59.02	F: 55.00 R: 50.00
*SREBP1C*	NM_001444604.1	F: TTTCTGACCCGCTTCTTCCT R: ACGGAACAACTGAGTCACCT	213	F: 58.94 R: 58.88	F: 50.00 R: 50.00
*DGAT2*	XM_021082452.1	F: CCCTCATAGCTGCCTACTCC R: GAGGAAAGACAGGACCCACT	155	F: 59.03 R: 58.65	F: 60.00 R: 55.00
*SCD*	NM_213781.1	F: CTTCCTGATCATTGCCAACA R: GCAAACCACCCTTCTCTTTG	191	F: 56.01 R: 57.20	F: 45.00 R: 50.00
*FASN*	NM_001099930.1	F: CTGATCAAGGTGCTGCTGTC R: CGAAGGAGTTTATGCCCACG	160	F: 58.91 R: 58.99	F: 55.00 R: 55.00
*FABP4*	NM_001002817.1	F: AAGAAGTGGGAGTGGGCTTT R: TTCCTGGCCCAATTTGAAGG	146	F: 59.15 R: 58.36	F: 50.00 R: 50.00
*18S*	NR_046261.1	F: GTAACCCGTTGAACCCCATT R: CCATCCAATCGGTAGTAGCG	151	F: 58.09 R: 57.93	F: 50.00 R: 55.00

## Data Availability

The original contributions presented in this study are included in the article/Supplementary Material. Further inquiries can be directed to the corresponding author(s).

## Supplementary Materials

The following supporting information can be downloaded at: https://www.mdpi.com/article/10.3390/cells15080684/s1, Figure S1: Western blotting analysis of PREF1, APN, FBP4, PPARγ and CEBPα in Porcine Preadipocytes.

## Abbreviations

The following abbreviations are used in this manuscript:

SFM-1	serum-free differentiation medium 1
SFM-2	serum-free differentiation medium 2
SFM-3	serum-free differentiation medium 3
OA	oleic acid
TRF	transferrin
INS	insulin
DEX	Dexamethasone
PA	Pantothenate
IBMX	3-Isobutyl-1-methylxanthine
RSG	Rosiglitazone
