# Supplementary material for: Oleic Acid and Transferrin Synergistically Induce Serum-Free Adipogenic Differentiation of Porcine Preadipocytes via the SEPTIN4/PPARγ Axis
Source: Cells. 2026 Apr 13;15(8):684. doi: 10.3390/cells15080684 (PMC13114657; doi:10.3390/cells15080684)
Supplement: Supplementary file 1 [file cells-15-00684-s001.zip › cells-4246310-supplementary.pdf]

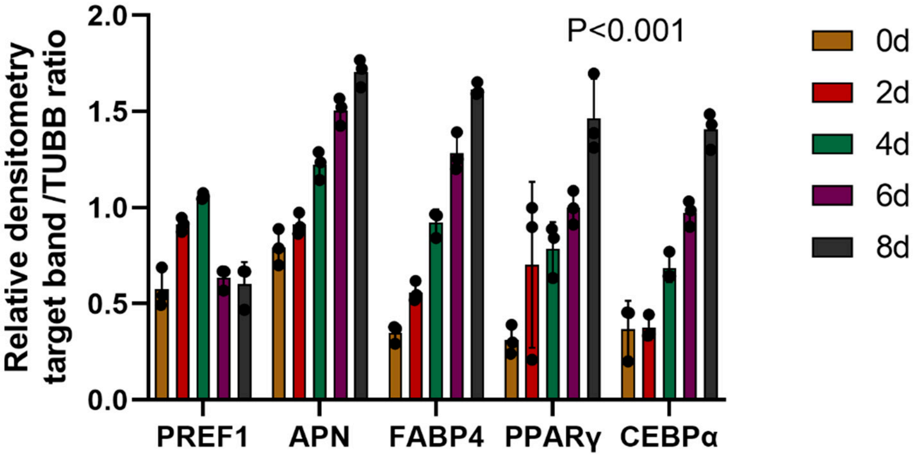

Supplementary Figure S1. Western blotting analysis of PREF1, APN, FBP4,PPARγ and CEBPα in Porcine Preadipocytes (n = 3 samples per group), ***p < 0.001
